# Impaired apoptosis of megakaryocytes and bone marrow mononuclear cells in essential thrombocythemia: correlation with *JAK2V617F* mutational status and cytoreductive therapy

**DOI:** 10.1007/s12032-012-0202-3

**Published:** 2012-03-15

**Authors:** Jacek Treliński, Krzysztof Chojnowski, Barbara Cebula-Obrzut, Piotr Smolewski

**Affiliations:** 1 Department of Hematology, Copernicus Memorial Hospital, Medical University of Lodz, ul Ciolkowskiego 2, 93-510 Lodz, Poland; 2 Department of Experimental Hematology, Copernicus Memorial Hospital, Medical University of Lodz, ul Ciolkowskiego 2, 93-510 Lodz, Poland

**Keywords:** Essential thrombocythemia, Apoptosis, Anagrelide, Hydroxyurea, *JAK2V617F* mutation

## Abstract

Essential thrombocythemia (ET) is a clonal myeloproliferative disorder characterized by overproduction of megakaryocytes (MKCs) and platelets. The recent discovery of the *JAK2* mutation has shed a new light on the development of ET but its pathogenesis still remains unknown. One of the possible mechanisms can be deregulation of apoptosis, resulting in accumulation of bone marrow MKCs. In this study, we investigated the apoptotic profile, as well as the expression of apoptosis-regulating protein in MKCs and bone marrow mononuclear cells (BMMCs) in 43 patients with ET. We found significantly lower percentages of apoptotic MKCs and BMMCs, as measured by the rate of annexin-V+ and caspase-3+ (Cas-3+) cells in relation to healthy volunteers. Additionally, the expression of Bax protein in ET patients naïve to cytoreductive treatment, as well as their Bax/Bcl-2 ratio, was significantly lower than in controls (*p* = <0.05 and *p* < 0.001, respectively). Patients positive for the *JAK2V617F* mutation had markedly higher activation of Cas-3, as well as higher Bax expression (*p* = 0.02 and *p* = 0.04, respectively) than *JAK2V617F* negative cases. There were no marked differences between patients already treated with anagrelide (ANA) or hydroxyurea (HU), although tendency toward the higher apoptosis rate was observed in the HU-treated group. In conclusion, these results demonstrate the inhibition of caspase-dependent apoptosis of both MKCs and BMMCs in untreated ET. This is associated with upregulation of Bcl-2 and downregulation of Bax proteins, predominantly in *JAK2V617F* negative cases. Patients treated with HU showed slightly higher pro-apoptotic Bax/Bcl-2 index than patients on ANA therapy, which may influence the better efficacy of HU therapy in ET.

## Introduction

Essential thrombocythemia (ET) is a clonal malignancy characterized by the excessive proliferation of megakaryocytes (MKCs) in the bone marrow and increased production of platelets. One of the possible mechanisms involved in pathogenesis of ET is deregulation of apoptosis, which results in accumulation of MKCs. Cellular defects that prevent apoptosis can result in the development of different hematological malignancies, including lymphoproliferative diseases. The classical example of the key role of inhibition of this process in pathogenesis of neoplasms is follicular lymphoma, in which the primary oncogenic change is the overexpression of Bcl-2 [1, 2].

The impaired apoptosis, with overexpression of anti-apoptotic genes and anti-apoptotic proteins, was previously described also in myeloproliferatine neoplasms, such as chronic myelogenous leukemia and primary myelofibrosis [3, 4]. It has also been demonstrated that peripheral blood mononuclear cells from patients with ET show resistance to apoptosis inducers while bone marrow haematopoietic progenitor CD34 cells overexpress mRNA for Fas, FAIM or *c*-FLIP proteins [5].

The aim of this study was to determine some parameters of apoptosis of MKCs and bone marrow mononuclear cells (BMMCs) in two groups of patients with ET: those naïve to treatment and those receiving standard cytoreductive therapy with either angrelide (ANA) or hydroxyurea (HU). Correlations between studied apoptosis markers and *JAK2V617F* mutational status were assessed. Attempts were also made to determine the influence of particular cytoreductive drugs on MKC and BMMC apoptosis in ET patients.

## Materials and methods

### Patients

Forty-three patients with ET were enrolled to the study after giving their informed consent. The study was approved by the local ethics committee. ET was diagnosed according to the World Health Organization 2008 criteria [6]. Twenty-two patients were previously untreated while 21 patients were on cytoreductive treatment (10 ANA, 11 HU). In the ANA or HU groups, measurements were only performed when the platelet count was below or equal to 400 × 10^9^/l (complete response) or <600 × 10^9^/l (partial response) after at least 4 weeks of treatment. The average dose of ANA was 1.5 mg/day (range, 1–4 mg/day) while the typical dose of HU was 1,000 mg/day (range, 500–2,000 mg/day). The control group consisted of 15 healthy subjects.

### Megakaryocyte detection

**Fig. 1:**
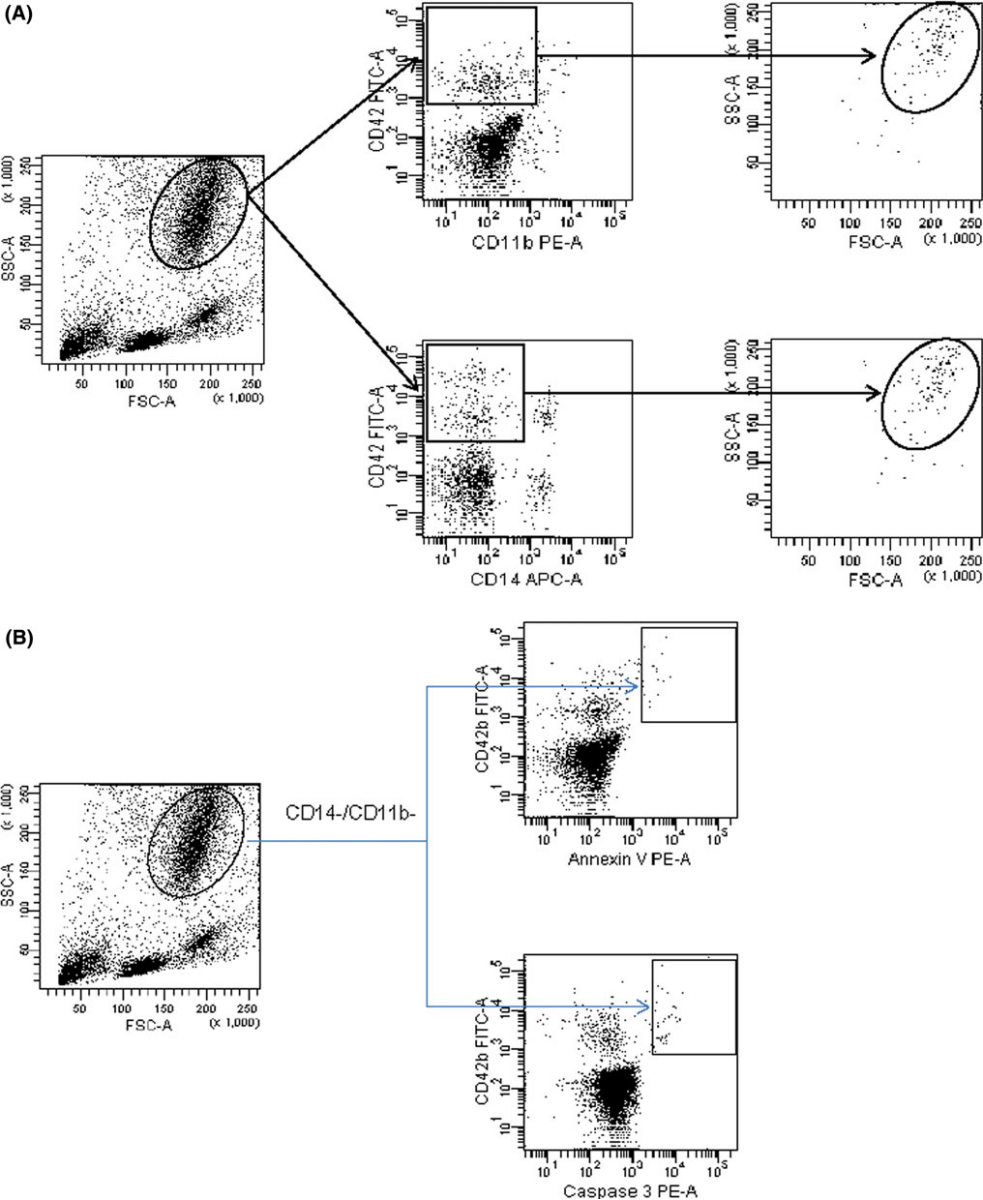
**a** Cytometric assessment of megakaryocytes (MKCs) based on forward scatter (FSC) versus side scatter (SSC) distribution. For excluding of monocyte-platelet and granulocyte-platelet conjugates, staining for CD42b, CD14 and CD11b was performed. MKCs were detected in FSC^high^/SSC^high^ population. **b** Detection of apoptosis parameters (Annexin-V, Caspase 3) on MKCs determined as FSC^high^/SSC^high^/CD42b+/CD14-/CD11b- cells

**Fig. 2:**
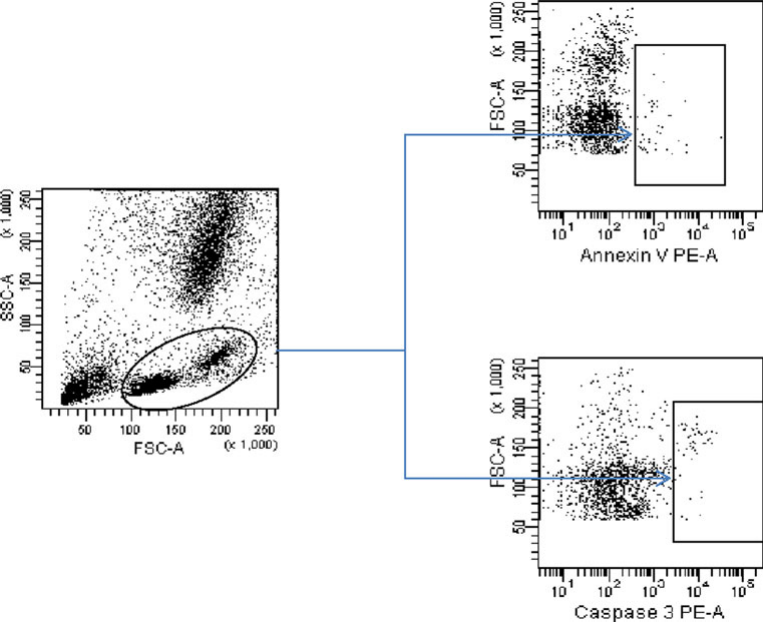
Detection of apoptosis parameters (Annexin-V, Caspase 3) on bone marrow mononuclear cells (BMMCs). BMMCs were defined based on FSC versus SSC distribution

In all patients, the percentages of MKCs and BMMCs were assessed. MKCs were detected in the whole bone marrow samples, based on forward scatter (FSC) versus side scatter (SSC) distribution, with expression of MKC-specific antigens. First, to exclude the monocyte-platelet and granulocyte-platelet conjugates, staining for CD14 and CD11b antigens using monoclonal antibody (MoAb) anti-CD14 (phycoerythrin-conjugated) and anti-CD11b [APC (allophycocyanin)-conjugated] was performed. Expression of CD42b antigen was assessed using fluorescein isothiocyanate (FITC)-conjugated anti-CD42b (all MoAbs from BD Pharmingen, San Jose, CA, USA). Based on this analysis, a high FSC (FSC^high^) and high SSC (SSC^high^) cells with expression of CD42b antigen were gated for apoptosis parameters (Fig. 1a, b). Additionally, in the series of primary experiments, these FSC^high^/SSC^high^/CD42b+ cell fractions highly co-expressed with another MKC marker, the CD61 antigen (BD Pharmingen, San Jose, CA, USA) (*r* = 0.95, *p* < 0.001). Finally, MKC were defined as a FSC^high^/SSC^high^/CD42b+/CD14−/CD11b-cells. BMMCs were discriminated based on FSC versus SSC distribution, as shown in Fig. 2.

### Assessment of apoptosis

The Annexin-V (Ann-V) staining and activated caspase-3 (Cas-3) assays were used as hallmarks of apoptosis, using commercially available kits from Becton–Dickinson, San Jose, CA, USA.

#### Ann-V assay

The rate of apoptosis was determined by the Ann-V method. In brief, after immunophenotyping and “lyse—no wash” procedure, the bone marrow cells were washed twice with cold phosphate buffered saline, PBS (Sigma Aldrich Chemie Gmbh, Germany) and then resuspended in 100 μl of binding buffer, containing 2 μl of R-phycoerythrin (R-PE)-conjugated Ann-*V.S.* The samples were then incubated for 15 min, at room temperature, in the dark. The fluorescence was measured immediately after staining by flow cytometry (FACScan; Becton–Dickinson, San Jose, CA, USA).

#### Active Cas-3 detection

Active Cas-3 was detected using FITC-conjugated monoclonal rabbit anti-active Cas-3 antibody (BD Pharmingen, San Diego, CA, USA). After immunophenotyping and the “lysed—not washed” procedure, the cells were fixed and permeabilized using Cytofix/CytopermTM (BD Pharmingen, San Diego, CA, USA) solution (20 min, on ice), then washed twice and resuspended in the Perm/WashTM buffer (BD Pharmingen, San Diego, CA, USA). The antibody was added at a concentration of 60 μl per 300 μl of cell suspension (30 min incubation, at RT). The fluorescence was measured directly after staining and washing in Perm/WashTM buffer by flow cytometry.

For further analysis, the FSC^high^/SSC^high^/Ann+/42b+ status was considered as a marker of MKC apoptosis. Also, the percentage of FSC^high^/SSC^high^/42b+/Cas-3+ cells was assessed for confirmation of caspase-dependent apoptosis.

### Assessing of apoptosis-regulating protein expression

In addition, the cellular expression of apoptosis-regulating proteins Bcl-2 and Bax in bone marrow mononuclear cells was assessed by flow cytometry. Freshly isolated bone marrow cells were fixed (as above) and additionally permeabilized with 0.1% polysorbate 20 (Tween-20) in PBS (Sigma Aldrich Chemie Gmbh, Germany). Mouse anti-human FITS-conjugated MoAbs anti-Bcl-2 (clone DO-7, DAKO, Glostrup, Denmark) was used at concentrations of 1:30 and 1:15, respectively, with the incubation time of 30 min, at room temperature, in the dark. Anti-Bax primary anti-human Ab (DAKO, Glostrup, Denmark) was used at a concentration of 1:400 (60 min. RT). Secondary swine anti-rabbit or anti-mouse Abs were used at concentrations of 1:20–1:50, where appropriate (30 min incubation, RT, in the dark). All antibody dilutions were made in 1% PBS-BSA. An elevation or decrease in expression of studied proteins in relation to control samples, measured as mean fluorescence intensity (MFI), was regarded as an indication of raised or lowered protein expression.

### Detection of JAK2-V617F

Genomic DNA was extracted from blood leukocytes using a commercial KIT (CHEMAGEN, Germany). The polymerase chain reaction (PCR) was performed in a total volume of 25 μl. Samples of 100 ng genomic DNA template and 50 pM of each primer were used. The oligonucleotide sequences (5’ to 3’) for RFLP (restriction fragments length polymorphism) of V617F were as described [7]. The 460-bp PCR product was digested with BsaXI (New England BioLabs, UK) for 3 h 37°C. The normal allele was digested into 3 fragments of 241, 189 and 30 bp, whereas the mutant-type remained undigested. The PCR denaturation was performed at 94°C for 30 s and annealing at 57°C for 30 s, with extension at 72°C for 30 s with a 30-cycle amplification. The digestion product bands were separated on 6% polyacrylamide gel, treated with ethidium bromide and visualized in ultraviolet (UV) light.

### Statistical analysis

Data are presented as medians and ranges for continuous variables and as count and percentage for categorical variables. Statistical comparisons between untreated ET, ET/ANA, ET/HU and control groups were performed using the Kruskal–Wallis ANOVA and median test for multiple independent samples. Statistical comparisons between two independent samples were made by Mann–Whitney U test. Correlations between variables were assessed by the Spearman rank correlation coefficient (*r*). In all tests, *p* values less than 0.05 were considered statistically significant. Statistical analysis was performed using STATISTICA *v.s.*8.0 (*Tulsa, OK, USA*) software.

## Results

The basic data of the patient and the control group are summarized in Table 1. Patients on both types of cytoreductive therapy demonstrated lower MKC expression in bone marrow than patients naïve to treatment, but the difference was not statistically significant.

**Table 1:** Characteristics of patients and control (Ctrl) group

Parameters	ET (*n* = 22)	ET/ANA (*n* = 10)	ET/HU (*n* = 11)	Ctrl (*n* = 15)
Sex M/F	17-May	8-Feb	7-Apr	9-Jun
Age (years)	62 (32–79)	57 (24–76)	61 (44–84)	61 (28–75)
PLT count (×10^9^/l)	780 (544–1,861)**	449 (258–595)	428 (332–581)	337 (207–402)
WBC count (×10^9^/l)	8.5 (5.3–20.0)*	8.4 (6.9–14.3)	6.4 (4.2–13.3)	6.8 (4.9–9.0)
MKCs (%)	26.1 (6.5–60.3)	15.9 (5.2–40.9)	16.1 (7.0–37.0)	13.2 (5.4–22.7)
Mutant *JAK2V617F* *n* (%)	10 (45)	4 (40)	8 (73)	

*MKCs* megakaryocytes, *ET* patients naïve to cytoreductive therapy, *ET/ANA* patients treated with anagrelide (ANA), *ET/HU* patients treated with hydroxyurea (HU)

* *p* < 0.05; ** *p* < 0.001 compared to Ctrl

The data concerning the apoptosis assessment are presented in Table 2. Significantly lower percentages of Ann-V+ MKCs were found in ET patients naïve to treatment and ET patients on ANA therapy in relation to the control group (*p* = 0.02 and *p* = 0.01, respectively). Consequently, the percentage of Cas-3+ MKCs was significantly lower in patients with ET at the time of diagnosis then in healthy volunteers (*p* = 0.03). Similarly, the BMMC rate of apoptosis was significantly higher in the control as compared to untreated patients with ET (Table 2). ET patients on HU therapy have a tendency toward higher spontaneous apoptosis of both types of cells, however, without statistical significance.

**Table 2:** Expression of studied parameters of apoptosis in ET and in the control (Ctrl) group

Parameters	ET (*n* = 22)	ET/ANA (*n* = 10)	ET/HU (*n* = 11)	Ctrl (*n* = 15)	Statistical significance
	Me (range)	Me (range)	Me (range)	Me (range)	
Ann-V+ MKCs (%)	1.7 (0.3–13.1)	1.5 (0.5–6.6)	3.9 (1.3–.8)	10.4 (1.5–30.5)	ET versus Ctrl**
					ET/ANA versus Ctrl*
Ann-V+ BMMCs (%)	2 (0.5–5.7)	1.3 (0.2–1.4)	1.9 (0.1–2.5)	3.26 (0.5–5.7)	ET versus Ctrl*
Cas3+ MKCs (%)	3.6 (0.2–22.7)	5.9 (0.3–9.4)	4.6 (2.4–7.4)	11.6 (2.9–42.5)	ET versus Ctrl*
Cas3+ BMMCs (%)	0.6 (0–1.7)	1.8 (0.1–2.5)	1.9 (0.2–3.4)	4.8 (0.2–14.1)	ET versus Ctrl**
BCL-2(MFI) in BMMCs	165.6 (31.2–861.2)	129.8 (38.6–197.6)	62.7 (24.6–254.3)	91.8 (47.4–157.3)	ns
BAX (MFI) in BMMCs	258.7 (67.8–930.7)	292.5 (73.9–535.2)	151.7 (44.9–253.9)	681.3 (407.2–1,041)	ET versus Ctrl**
					ET versus ET/HU*
					ET/ANA versus Ctrl*
					ET/HU versus Ctrl**
BAX/BCL-2 index in BMMCs	1.96 (0.27–5.27)	2.34 (1.42–7.04)	2.49 (0.85–2.98)	9.08 (2.59–19.0)	ET versus Ctrl**
					ET/ANA versus Ctrl*
					ET/HU versus Ctrl*

*MKCs* megakaryocytes, *BMMCs* bone marrow mononuclear cells, *ET* patients naïve to cytoreductive therapy, *ET* *+* *ANA* patients treated with anagrelide (ANA); *ET* *+* *HU* patients treated with hydroxyurea (HU), *Me* median, *MFI* mean fluorescence intensity

* *p* < 0.05; ** *p* < 0.001; *ns* no significant

The median values of Bax protein expression in MKCs were markedly higher in the control group than in all ET subgroups (control vs. ET, *p* = 0.004; control vs. ET/ANA, *p* = 0.03; control vs. ET/HU, *p* < 0.001). Additionally, significant difference in Bax expression between untreated patients and those treated with HU was found (*p* = 0.04). Bax/Bcl-2 index was distinctly higher in controls than in ET patients naïve to therapy (*p* < 0.001) as well in patients treated with ANA (*p* = 0.02) and HU (*p* = 0.03). The BMMCs of *JAK2V617F* positive patients demonstrated a significantly higher activation of Cas-3 (0.7 vs. 0.2, *p* = 0.04) as well as higher Bax expression (MFI, 380.3 vs. 209.12, *p* = 0.02) than those in *JAK2V617F* negative cases (Table 3). Statistically significant positive correlations between WBC and PLT (*r* = 0.43, *p* < 0.05) and Bax and Bcl-2 (*r* = 0.63, *p* = 0.04) were found in untreated ET patients. Interestingly, a negative correlation between age and Bax (*r* = −0.64, *p* < 0.03) was only seen in *JAK2V617F*+ cases. In patients treated with ANA, a significant positive correlation between Bax and Bcl-2 (*r* = 0.91, *p* = 0.001) was found, while in the HU-treated group, a positive correlation between Bax and Bcl-2 (*r* = 0.7, *p* < 0.05) was observed.

**Table 3:** Comparison of studied parameters of apoptosis in *JAK2V617F*-positive and *JAK2V617F*-negative ET untreated patients

Parameters	ET	ET	Statistical significance
	*JAK2V617F*+ (*n* = 10)	*JAK2V617F*− (*n* = 12)	
	Me (range)	Me (range)	
Ann-V+ MKs (%)	2.3 (0.3–13.1)	1.1 (0.5–7.7)	ns
Cas-3+ BMMCs (%)	0.7 (0.2–14.3)	0.2 (0.06–3.1)	*p* = 0.04
BCL-2 (MFI) in BMMCs	165.6 (31.2–353.1)	137.5 (35.6–861.2)	ns
BAX (MFI) in BMMCs	380.3 (103.0–930.7)	209.12 (67.8–693.8)	*p* = 0.02
BAX/BCL-2 index in BMMCs	2.32 (0.87–5.27)	1.31 (0.27–4.12)	ns

*ET* patients naïve to cytoreductive therapy, *Me* median, *MFI* mean fluorescence intensity, *ns* no significant

## Discussion

The impaired balance between the ability for cells to proliferate and undergo apoptosis may be crucial for the development of malignant hematological disorders including ET [1]. To the best of our knowledge, this is the first report showing inhibited apoptosis of ET MKCs in bone marrow as assessed by flow cytometry. We observed a markedly lower percentage of MKCs positive for both Ann-V staining and active Cas-3 in patients with ET naïve to therapy as compared to healthy volunteers. This is contrary to the results presented by Florena et al. [8] who found a similar percentage of Cas-3-expressing MKCs in ET versus controls, however, this study was based just on an immunohistochemical evaluation of bone marrow samples. This is an important distinction, because the flow cytometry method is a much more sensitive method of detecting a relatively small population of characteristic cells. Interestingly, cases with a low apoptotic fraction of Cas-3 (<5%) were associated with the occurrence of thrombotic events, which are still the main cause of morbidity and mortality in ET [8].

The actual fraction of bone marrow mononuclear cells committed to apoptosis was assessed by the Ann-V staining, with estimation of effector Cas-3 activation. Our study showed a markedly lower percentage of apoptotic MKCs and BMMCs in ET patients than in controls. Moreover, the Bax/Bcl-2 index was much lower in ET patients, comparing to the control.

Activation of caspases and other events during the process of apoptosis is regulated on multiple levels by Bcl-2 family members acting on the intrinsic pathway of apoptosis [9–11]. The Bcl-2 family consists of several proteins, with the most important members being the pro-apoptotic Bax and anti-apoptotic Bcl-2 proteins. The inhibited Bax/Bcl-2 ratio is one of the most sensitive markers of the anti-apoptotic cell profile. Moreover, it has been previously demonstrated that transcription of the anti-apoptotic Bcl-2 protein is regulated by the STAT3/5 signaling pathway and that Bcl-2 is overexpressed in erythroid cells from patients with polycythemia vera [12]. It has also been discovered that MKCs from patients with ET as opposed to primary myelofibrosis are characterized by elevated expression of Bcl-XL and markedly lower expression of pro-apoptotic Bax (8). Additionally, Zhang et al. [13] observed strong expression of Bcl-XL in immature MKCs and decreased expression in mature MKCs. They concluded that increased Bcl-XL expression may be essential to MKC maturation. In the present study, we found downregulation of Bax expression in bone marrow BMMC in patients with ET as compared to controls. This, together with the higher expression of Bcl-2 protein and markedly lower Bax/Bcl-2 index, confirms the anti-apoptotic profile of BMMCs in ET patients.

In 2005, a somatic activation mutation in Janus Kinase 2, occurring in approximately 50% of ET patients, was identified [14, 15]. The *JAK2V617F* gene mutation results in constitutive signaling through the *JAK2* tyrosine kinase, leading to increased cellular proliferation. On the other hand, it has been speculated that the constitutive activation of *JAK2* could protect cells from death receptor-induced apoptosis by different means, in this number by upregulation of the anti-apoptotic gene *c*-*FLIP* [16–19]. In our study, the cohort of *JAK2V617F* positive patients demonstrated a markedly higher activation of *Cas*-*3,* as well as higher Bax expression, and also a tendency toward increased apoptosis measured by Ann-V method as compared to *JAK2* negative cases. Also, the Bax/Bcl-2 ratio was shifted toward more pro-apoptotic in *JAK2*+ patients. Our observation can partially explain the phenomenon of increased efficacy of cytoreductive drugs in patients positive for *JAK2* mutation [20].

It has been stated repeatedly that not all patients with ET require therapy, unless certain risk factors (age >60 years, prior thrombotic or hemorrhagic event, cardiovascular diseases, possibly *JAK2V617F* mutation and elevated leukocytosis) are present [21–25]. HU and ANA are most widely used as first-line cytoreductive therapy in high-risk ET patients. HU has emerged as the treatment of choice in two randomized clinical trials [24, 25]. HU is an antimetabolite, mediating its anti-myeloproliferative effects via the inhibition of ribonucleoside diphosphate reductase needed for synthesis and repair of DNA [26]. Beyond its broad myelosuppressive activity, HU can also exert an antithrombotic activity by affecting polymorphonuclear leukocyte function and their interactions with platelets [27–29]. Recent studies have shown that HU can induce apoptosis in many types of cells: for example, endothelial cells, human mesenchymal stem cells and mouse embryonic stem cells [30, 31]. HU appears to promote cell death by regulating the expression levels of Bcl-2 and the tumor suppressor p53 protein [32, 33]. In contrast, ANA is a non-cytostatic imidazoquinazoline derivative, which reversibly disrupts MKC maturation (e.g., ploidy, size, cytoplasmic maturation) in a dose-dependent fashion by influencing the post-mitotic phases of MKC development [34]. There is no clear-cut evidence to suggest a role is played by ANA in reducing the total number of MKCs or stimulating apoptosis [35, 36]. In the present study, different markers of apoptosis were examined in 21 patients with ET treated with HU or ANA who were in complete or partial remission according to European Leukemia Net criteria [37]. Our analysis showed that, in relation to patients naïve to therapy, both ANA and HU downregulated concentration of anti-apoptotic Bcl-2 while HU, surprisingly, downregulated the concentration of pro-apoptotic Bax. However, it has to be stressed that the overall Bax/Bcl-2 ratio in patients on ANA or HU therapy was more pro-apoptotic than in patients at the time of diagnosis. There were no statistical differences in other studied parameters although there was a tendency toward a higher percentage of Ann-V+ MKc and Cas-3+ BMMCs in the HU group.

In conclusion, we found evidence for caspase-dependent MKC and BMMC apoptosis inhibition with upregulation of Bcl-2 protein and concurrent downregulation of Bax in ET patients in relation to healthy volunteers. Patients positive for the *JAK2V617F* mutation had a tendency toward increased apoptosis of MKCs and BMMCs in bone marrow in relation to *JAK2V617F* negative cases, which could contribute to the better therapy outcome observed in this group of patients. Patients treated with HU showed a slightly higher pro-apoptotic Bax/Bcl-2 index than patients on ANA therapy, although no significant differences in apoptosis markers were observed between these treatment modalities.
